# Xiaoketongbi Formula vs pregabalin for painful diabetic neuropathy: A single‐center, randomized, single‐blind, double‐dummy, and parallel controlled clinical trial

**DOI:** 10.1111/1753-0407.13306

**Published:** 2022-08-30

**Authors:** Qiyun Lu, Benjian Chen, Qingshun Liang, Liyan Wu, Lulu Luo, Anxiang Li, Wenwei Ouyang, Zehuai Wen, Yunwei Liu, Jiayan Lu, Yunyi Liu, Guanjie Fan, Zhenjie Liu

**Affiliations:** ^1^ Guangdong Provincial Hospital of Chinese Medicine Guangzhou China; ^2^ The Second Affiliated Hospital of Guangzhou University of Chinese Medicine Guangzhou China; ^3^ Guangzhou University of Traditional Chinese Medicine Guangzhou China; ^4^ Guangzhou Medical University Guangzhou China

**Keywords:** Chinese herbal medicine, painful diabetic neuropathy, pain therapy, pregabalin, 中草药, 疼痛疗法, 痛性糖尿病神经病变, 普瑞巴林

## Abstract

**Background:**

We assessed the efficacy and safety of the Xiaoketongbi Formula (XF) vs. pregabalin in patients with painful diabetic neuropathy (PDN).

**Methods:**

Patients with PDN (n = 68) were included in a single‐center, randomized, single‐blind, double‐dummy, parallel controlled clinical trial. The primary outcome was the change in the Brief Pain Inventory for Diabetic Peripheral Neuropathy (BPI‐DPN). Secondary outcomes evaluated included the reduction of BPI‐DPN >50%, changes in the numeric rating scale‐11 (NRS‐11) score for pain, Daily Sleep Interference Diary (DSID), Patient Global Impression of Change (PGIC), nerve conduction velocity (NCV), and adverse events.

**Results:**

After 10 weeks of treatment, the BPI‐DPN score reduced from 42.44 ± 17.56 to 26.47 ± 22.22 and from 52.03 ± 14.30 to 37.85 ± 17.23 in the XF and pregabalin group (*P*_s_ < 0.001), respectively. The difference in the absolute change in BPI‐DPN score between both groups was −1.79 (95% CI: −9.09, 5.50; *p* = 0.625). In the XF and pregabalin groups, 44.1% (15/34) and 20.6% (7/34) of patients reported a BPI‐DPN reduction >50% (*p* = 0.038), respectively. There were no significant differences between groups in NRS‐11 and DSID (*P*_s_ > 0.05). A significantly greater number of patients in the XF group felt “significantly improved” or “improved” than in the pregabalin group (35.3% (12/34) vs. 11.8% (4/34), *p* = 0.045). The absolute change in motor nerve conduction velocity of the right median nerve was significantly different between both groups (XF group 0.7 ± 2.3 vs. pregabalin group −2.2 ± 4.1, *p* = 0.004). No serious adverse events were reported in either group.

**Conclusions:**

XF is equivalent to pregabalin in reducing pain symptoms and improves the quality of life in patients with PDN. In addition, XF has the potential to improve nerve function by increasing NCV.

## Graphical abstract

**Highlights**

- This is the first randomized, single‐blind, double‐dummy, parallel controlled clinical trial collecting data on the efficacy and safety of Chinese herbal medicines compared to that of pregabalin in patients with painful diabetic neuropathy.
- The Xiaoketongbi Formula is an effective treatment that can relieve pain symptoms, improve quality of life, and potentially improve nerve conduction velocity in patients suffering from painful diabetic neuropathy.

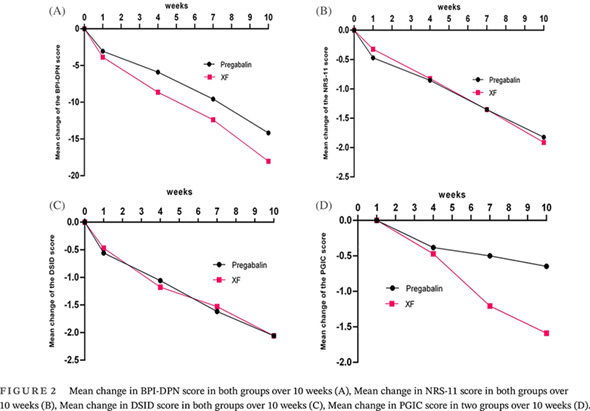

## 摘要

**目的:**

比较消渴通痹方(XF)与普瑞巴林治疗痛性糖尿病神经病变(PDN)的疗效和安全性。

**方法:**

对68例PDN患者进行单中心, 随机, 单盲, 双模拟, 平行对照临床试验。主要结果是糖尿病周围神经病变的简明疼痛问卷(BPI‐DPN)的变化。次要结果包括BPI‐DPN下降>50%, 疼痛数字评定量表‐11(NRS‐11)评分的变化, 日常睡眠干预日记(DSID), 患者总体变化印象(PGIC), 神经传导速度(NCV)和不良事件。

**结果:**

治疗10周后, XF组和普瑞巴林组的BPI‐DPN评分分别从42.44±17.56降至26.47±22.22和52.03±14.30降至37.85±17.23(P<0.001)。两组间BPI‐DPN评分绝对值变化差异为‐1.79(95%CI:‐9.09, 5.50, P=0.625)。XF组和普瑞巴林组分别有44.1%(15/34)和20.6%(7/34)的患者报告BPI‐DPN下降>50%(P=0.038)。两组间NRS‐11和DSID评分差异无统计学意义(P>0.05)。与普瑞巴林组相比, XF组有显著改善或改善的患者数量显著增加(35.3%(12/34)比11.8%(4/34), P=0.045)。两组右侧正中神经运动传导速度绝对值变化差异有统计学意义(XF组0.70±2.3vs.普瑞巴林组‐2.2±4.1, P=0.004)。两组均未报告严重不良事件。

**结论:**

XF在减轻PDN患者疼痛症状, 提高生活质量方面与普瑞巴林相当。此外, XF还有可能通过增加神经传导速度来改善神经功能。

## 1 INTRODUCTION

Diabetic peripheral neuropathy (DPN) is one of the most common complications of diabetes. Painful diabetic neuropathy (PDN) is a special type of DPN, with a prevalence rate of 11.0% to 53.7% in diabetic patients, which seriously affects physical and mental health and increases the economic burden on patients.^1^ The pathogenesis of PDN is widely thought to be related to factors such as hyperglycemia, open aldose reductase‐polyol‐inositol pathway, and abnormal protein glycosylation.^2^ Notwithstanding that blood glucose control forms the basis of all diabetes treatments, researchers found that strict control of blood glucose in type 2 diabetic patients has limited efficacy in PDN.^3^, ^4^, ^5^, ^6^ PDN manifests as spontaneous burning or stabbing type of pain at the distal ends of the extremities accompanied by hypersensitivity or hypoesthesia, exhibiting a symmetrical pattern. Pain is often aggravated at night, seriously affecting sleep and triggering depression and anxiety in patients. Moreover, PDN affects the physical and mental health of patients. Compared with painless diabetic neuropathy, PDN is known to have an obvious negative effect on quality of life, both physically and mentally.^7^ The “Guidelines for Diabetic Neuropathy Pain Management” issued by the American Academy of Neurology recommend assessing the degree of pain relief and improvement of patient physical function and quality of life when evaluating the effect of PDN treatment. Therefore, identifying novel approaches to relieve pain and improve sleep and the quality of life of patients is key in treating PDN.

Pain management is important in the treatment of PDN. Pregabalin is currently the most thoroughly studied drug and is recommended in international guidelines as the first‐line drug for treating PDN.^8^, ^9^, ^10^ Overwhelming evidence substantiates that pregabalin can relieve pain in at least 30%–50% of PDN patients.^11^, ^12^ According to published reports,^13^, ^14^, ^15^, ^16^ pregabalin can improve sleep, relieve anxiety, and generally improve quality of life as manifested in a combination of pain relief, improved sleep, and direct effects on patient function.^13^ However, the clinical applications are limited because of its various adverse reactions, including a possible increase in suicidal thoughts and behaviors. Therefore, there is an urgent need to find a safe and effective alternative therapy for treating PDN.

Chinese herbal medicines (CHMs) have a wide range of pharmacological effects and play an increasingly important role in treating diseases with complex pathogenesis and symptoms like PDN. There is ample evidence^15^, ^17^, ^18^, ^19^ suggesting that CHMs can relieve pain associated with PDN, and they are commonly used in China. However, there have been few prospective randomized controlled trials, and most of these studies used a placebo as the control. Moreover, there are as yet no published clinical studies comparing the efficacy of CHMs with anodyne (recommended by current guidelines).^20^

The Xiaoketongbi Formula (XF) is a CHM originating from the historical Tao‐He‐Cheng‐Qi Decoction. The biological activity of the components of XF was ascertained based on hundreds of medical records for effective treatment of PDN from Guangdong Provincial Hospital of Chinese Medicine, a tertiary hospital in southern China. Peach kernel, *Rheum officinale*, *Astragalus mongholicus*, *Angelica sinensis*, and *Salvia* are the main components of XF.

Pharmacological studies found that astragaloside IV, the main active component of Astragali Radix, has neuroprotective effects and promotes neovascularization.^21^, ^22^ Moreover, *A. sinensis* can promote the recovery of neuron structure and function and increase nerve conduction speed.^23^ Researchers have shown that salvianolic acid A in *Salvia* relieves the symptoms of PDN by increasing the pain threshold and nerve conduction velocity (NCV).^24^ Moreover, amygdalin sourced from peach kernels stimulates the growth of neurites and has neurotrophic activity.^25^ In addition, the antioxidant effects of resveratrol and emodin in rhubarb can reportedly reduce nerve cell damage.^26^, ^27^

Based on observations from hundreds of previously unpublished clinical cases, we believe that XF may effectively relieve diabetic neuropathic pain. This study sought to assess the efficacy and safety of XF in a randomized controlled trial and evaluate whether XF (in comparison with pregabalin) can relieve neuropathic pain and improve quality of life for patients with PDN.

To our knowledge, this is the first randomized, single‐blind, double‐dummy, parallel controlled clinical trial in which the efficacy and safety of CHMs were compared to that of pregabalin in patients with PDN. Our results broaden the therapeutic landscape for patients with PDN. In China, pregabalin is the first‐line drug, recommended in Chinese guidelines, for treating PDN.^28^ For ethical reasons, a placebo could not be used as a control for many PDN patients due to the severity of the pain; accordingly, pregabalin was selected as the medication for the control group.

## 2 RESEARCH DESIGN AND METHODS

### 2.1 Study design and patients

This was a single‐center, randomized, single‐blind, double‐dummy, parallel controlled clinical trial carried out from August 1, 2018 to December 30, 2020 at the Guangdong Provincial Hospital of Chinese Medicine in China. The lead‐in period was 4 weeks, and the treatment period was 10 weeks.

The patients included in the study had been diagnosed with type 2 diabetes, based on the 1999 World Health Organization diagnostic criteria.^29^ All the study participants presented with distally distributed neuropathic pain and had been diagnosed with DPN based on a structured clinical examination. The additional inclusion criteria were: (1) age ranging from 18–70 years, (2) numerical rating scale‐11 (NRS‐11) score ≥4, (3) patient had no history of treatment for PDN or received PDN treatment and completed the required wash‐out, and (4) patient provided written informed consent.

The main exclusion criteria were: The main exclusion criteria were:

1. neuropathy caused by other diseases: cervical and lumbar spine disease, cerebral infarction, Guillain‐Barré syndrome, severe arteriovenous vascular disease, neurotoxic effects caused by drugs, etc.
2. severe heart, liver, or kidney insufficiency (cardiac function grade III and above, alanine aminotransferase ≥ 2.5 times and/or total bilirubin ≥1.5 times the upper limit of normal, estimated glomerular filtration rate (eGFR) <30 mL/min.
3. patients with malignant tumors.
4. psychiatric patients.
5. pregnant and breastfeeding women.
6. diabetes with acute complications and concurrent infections in the past one month.
7. allergic constitution or a history of allergies to the drugs in this study.
8. concomitant treatment with other medications for pain management.

Ensuring patient compliance was essential to obtain accurate study findings. We fully communicated with patients before the trial to obtain patient trust. Patients were informed that treatment would take time to work. Furthermore, the chronic disease management model was adopted in this study, and a full‐time staff person was responsible for the follow‐up management of patients, with the aim of reducing patient dropout.

The study protocol was approved by the Institutional Ethics Committee of Guangdong Provincial Hospital of Chinese Medicine (approval ref 2018‐098‐01) and registered at the Chinese Clinical Trial Registry (Registration Number ChiCTR1800019046). All participants provided written informed consent before randomization.

### 2.2 Randomization and treatment

Participants were assigned to the intervention or control group at a ratio of 1:1 using the sequentially numbered, opaque, sealed envelope (SNOSE) technique. The envelopes were designated as “intervention” or “control” by randomization, with random numbers generated from statistical software. Each participant received a sealed envelope with information indicating their assigned group.

Only the investigators knew which patients were assigned to which group; the patients, supervisors, outcome assessors, and statisticians were blinded to the allocation. Masking for patients was achieved by a double‐dummy placebo design. Pregabalin was produced by Pfizer Manufacturing Deutschland GmbH, Betriebsstätte Freiburg, production batch J20160021. The placebo of pregabalin was produced by Boji Pharmaceuticals Biotechnology Co Ltd, which was matched to pregabalin in appearance, color, and taste.

The XF herbs were extracted with hot water, concentrated, spray‐dried, processed into granules, and packed in sealed opaque sachets. Production was controlled rigorously according to good manufacturing practice standards by Huarun Sanjiu Pharmaceuticals Co Ltd. The same manufacturer produced the XF placebo, using a 0% test drug, caramel pigment, bittering agent, and dextrin. The XF placebo was similar in color, smell, taste, appearance, and packaging to the XF granule.

Participants in the XF group were instructed to dissolve 15 g of XF granules in 150 ml of boiled water and to take this solution orally once daily as well as the pregabalin‐matched placebo capsule once daily for the first week and twice daily from the second week until the end of the study. In the pregabalin group, the participants were instructed to take 75 mg pregabalin orally once daily for the first week and 75 mg twice daily from the second week until the end of the study, along with 15 g of the XF‐matched placebo once daily for 10 weeks.

All participants received comprehensive diabetes management according to guidelines,^30^ including health education, diet, exercise, routine blood pressure lowering, and blood glucose and lipids control. Any treatment of PDN and drugs that affect the test results or CHMs (other than the study medication) were prohibited during the trial.

Patients were examined at baseline and on weeks 1, 4, 7, and 10. At each visit, the fasting blood glucose levels and blood pressure were measured and scores obtained for the Brief Pain Inventory for Diabetic Peripheral Neuropathy (BPI‐DPN), NRS‐11, Daily Sleep Interference Diary (DSID), and Patient Global Impression of Change (PGIC). Safety indices and NCV were measured at the baseline time point and at the end of the study.

### 2.3 Outcomes and definitions

The primary outcome was the difference between the two groups in terms of the absolute change of the BPI‐DPN after treatment for 10 weeks. The BPI‐DPN is a patient‐completed numeric rating scale that assesses the severity of pain and its impact on daily functioning, which has been specifically validated for PDN.^31^, ^32^ The four comparative descriptors of severity of pain were: “worst”, “least”, “average”, and “now” (current) pain on an 11‐point numeric rating scale (NRS) from zero for “no pain” or “does not interfere” to 10 for “the most pain” or “completely interferes”. The interference items included daily activities, mood, walking ability, daily work (work outside the home and housework), relationship with others, sleep, and life interests.

The secondary outcomes included the reduction of BPI‐DPN >50% and absolute changes in the scores of NRS‐11, DSID, PGIC, and NCV at 10 weeks compared with baseline. For NRS‐11, a score of 0 indicates no pain, and a score of 10 indicates the highest pain level. DSID was used to record the impact of the patient's pain on sleep, with “0” signifying no effect on sleep at all and “10” signifying the complete inability to fall asleep because of pain. PGIC is the overall sensory self‐score of the patient after treatment, which is graded as: 1 (significantly improved), 2 (improved), 3 (slightly improved), 4 (no improvement), 5 (slightly worsened), 6 (remarkably worsened), to 7 (very bad). Lastly, NCV was completed via electromyography to measure the motor nerve conduction velocity (MNCV) and sensory nerve conduction velocity (SNCV) of the median, tibial, and sural nerves. The DEYPOINT electromyography machine (DANTEC, Denmark), using the surface electrode method, was applied in the examination room.

The participants were asked to report any symptoms and adverse events (AEs) at each follow‐up visit or immediately upon onset of the AE. In addition, levels of serum creatinine, aspartate aminotransferase, and alanine aminotransferase, as well as blood cell counts, were checked as safety indices.

### 2.4 Data analysis and statistics

A reduction in BPI‐DPN level after treatment was the primary outcome. The sample size was estimated using a two‐sample means superiority test and comparison. According to the literature,^33^ it was assumed that after the intervention, pregabalin could reduce BPI‐DPN by 13.2 ± 13.1, and XF could reduce BPI‐DPN by 27.3 ± 20.0. The PASS 11.0 software calculated the sample size with α = .05 and a power of 0.9. The sample size required for each group was 29. With a dropout rate of 15%, the total number of patients was 68, divided into two equal groups: 34 in the XF group and 34 in the pregabalin group.

The superiority test results were expressed by a two‐sided 95% CI of the difference between the groups. Descriptive statistics were generated for demographic data and baseline analysis. Continuous variables are presented as the mean, standard deviation, minimum, maximum, median, and interquartile range. Categorical variables are presented as frequency and percentage.

The primary and secondary outcomes were analyzed according to the intent‐to‐treat principle using the full analysis set (FAS). Descriptive analysis was performed for the proportion of patients with a reduction of BPI‐DPN >50%, and the between‐group difference was assessed using the chi‐square test. Changes in BPI‐DPN, NRS‐11, DSID, and PGIC from baseline were analyzed over time, and the between‐group differences were assessed using the *t* test or rank sum test. A paired *t* test was used to compare the pre‐ and posttreatment levels of various parameters.

The incidence rates of AEs and serious adverse events (SAEs) were calculated. Safety analyses were performed on the safety set.

All analyses were performed using the SPSS 18.0 software (IBM Corporation). A two‐sided *p* < 0.05 was considered statistically significant.

## 3 RESULTS

### 3.1 Baseline characteristics

**FIGURE 1:**
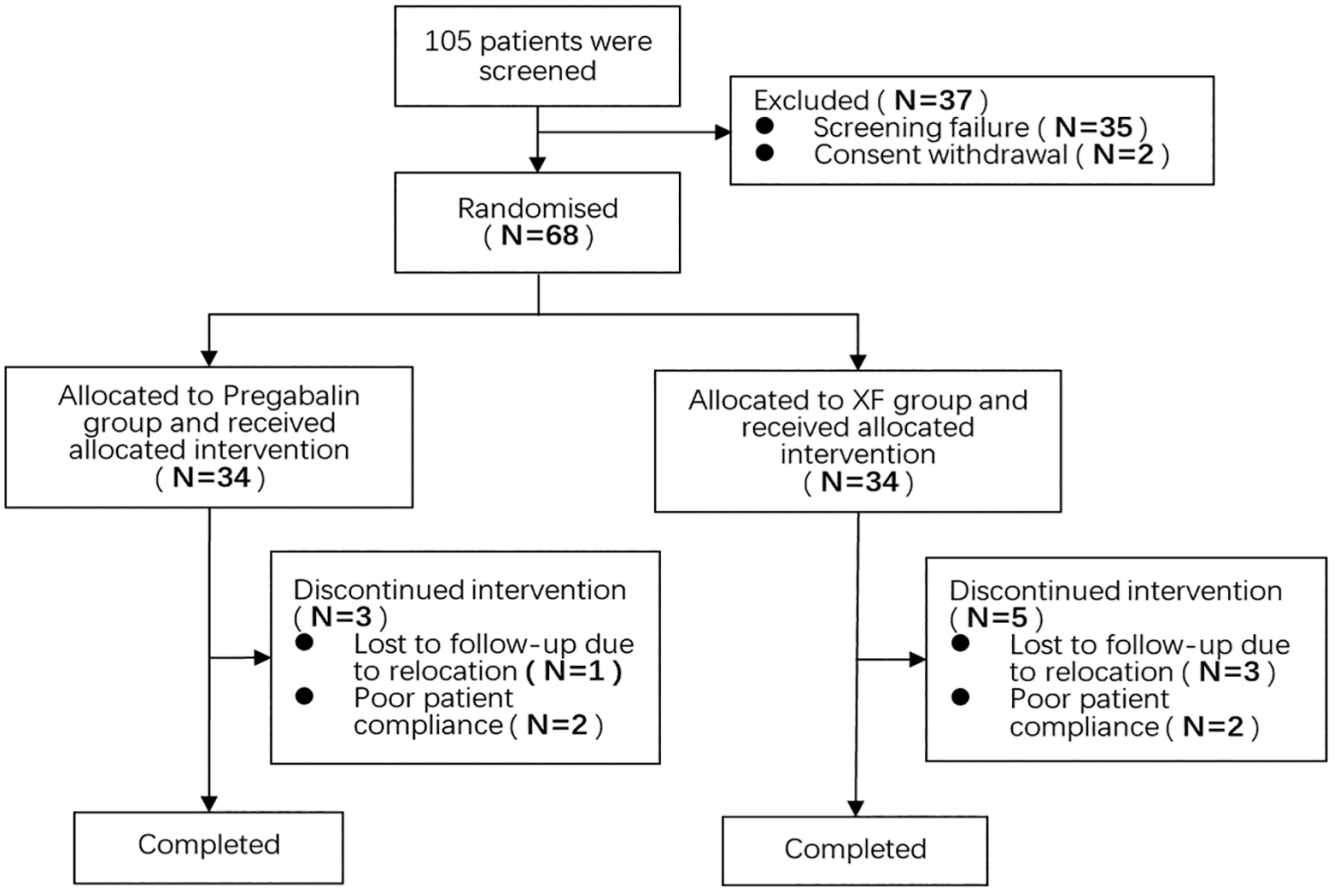
Participant screening flowchart.

Out of the 105 patients first registered in the study (prescreening), 68 fulfilled the enrollment criteria and were assigned at random to the XF group (n = 34) or pregabalin group (n = 34). Sixty‐eight participants were included in the FAS. Five patients in the XF group and three in the pregabalin group dropped out (Figure 1). A total of 60 patients completed the study. There were no statistically significant differences between the two groups in terms of gender, age, weight, history of tobacco and alcohol consumption, duration of diabetes, duration of PDN, fasting blood glucose, and glycosylated hemoglobin (*P*_s_ > 0.05) (Table 1).

**TABLE 1:** Baseline characteristics of the study participants (FAS population)

Characteristics	Pregabalin group (n = 34)	XF group (n = 34)	*p*
Male sex, n (%)	20 (58.8%)	21 (61.8%)	0.804
Age (y), mean ± SD	57.15 ± 10.45	55.29 ± 10.51	0.468
Weight (kg), mean ± SD	55.85 ± 11.77	57.43 ± 9.00	0.538
BMI (kg/m^2^), mean ± SD	20.76 ± 3.21	21.63 ± 3.04	0.258
Smoking (yes), n (%)	6 (17.6%)	10 (29.4%)	0.253
Alcohol (yes), n (%)	7 (20.6%)	5 (14.7%)	0.525
Duration of diabetes (mo), (p25 ~ p75)	42 (11.0 ~ 110.0)	48 (8.75 ~ 123)	0.927
Duration of pain (mo), (p25 ~ p75)	12 (4.0 ~ 34.5)	9 (5.5 ~ 25.0)	0.606
FBG (mmol/L), (p25 ~ p75)	6.8 (5.5 ~ 8.5)	6.0 (5.1 ~ 9.1)	0.228
HbA1c (%), (p25 ~ p75)	6.7 (4.0 ~ 14.5)	6.7 (5.9 ~ 9.7)	0.393
Anodyne (yes), n (%)	10 (29.4%)	7 (20.6%)	0.401
Neurotrophic (yes), n (%)	22 (64.7%)	22 (64.7%)	1.000
Aldose reductase inhibitor (yes), n (%)	6 (17.6%)	4 (11.8%)	0.493
Lipoic acid antioxidant (yes), n (%)	3 (8.8%)	0 (0%)	0.239
Insulin (yes), n (%)	11 (32.4%)	14 (41.2%)	0.451
OHD (yes), n (%)	25 (73.5%)	24 (70.6%)	0.787

Abbreviations: BMI, body mass index; FAS, full analysis set; FBG, fasting blood glucose; HbA1c, glycosylated hemoglobin; OHD, oral hypoglycemic drugs; p, median (interquartile range); XF, Xiaoketongbi Formula.

The prior treatments received in the two groups are presented in Table 1. There were no significant differences in baseline medications between the two groups (*p* > 0.05). Neither group underwent nonpharmacological therapies, such as transcutaneous electrical nerve stimulation, spinal cord stimulation, acupuncture, and infrared therapy.

Before treatment, 41.2% (14/34) of the XF group vs. 32.4% (11/34) of the pregabalin group received insulin. During treatment, 64.7% (22/34) of the XF group vs. 67.6% (23/34) of the pregabalin group received insulin. There was no significant difference in insulin treatment between the two groups (*p* = 0.789).

### 3.2 Changes in BPI‐DPN

**FIGURE 2:**
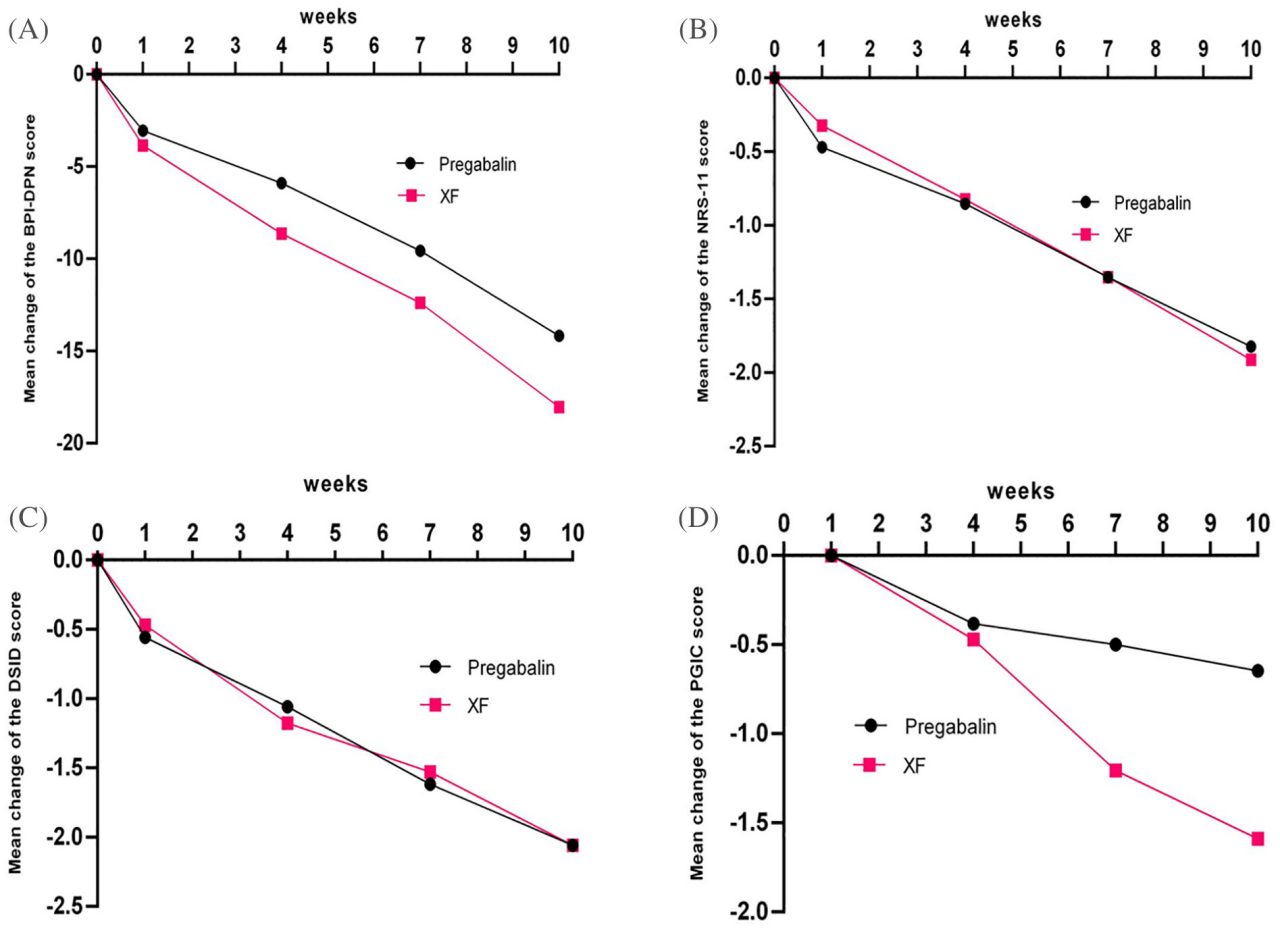
Mean change in BPI‐DPN score in both groups over 10 weeks (A), Mean change in NRS‐11 score in both groups over 10 weeks (B), Mean change in DSID score in both groups over 10 weeks (C), Mean change in PGIC score in two groups over 10 weeks (D).

The BPI‐DPN scores of both groups tended to decrease. After 10 weeks of treatment, the BPI‐DPN score was reduced from 42.44 ± 17.56 to 26.47 ± 22.22 in the XF group and from 52.03 ± 14.30 to 37.85 ± 17.23 in the pregabalin group (*P*_s_ < 0.001) (Table 2). The absolute change in the BPI‐DPN score was 15.97 ± 15.99 in the XF group and 14.18 ± 14.08 in the pregabalin group. There were no significant differences in absolute change of the BPI‐DPN; the between‐group difference was −1.79, and the lower bound of the 95% CI was −9.09 < 0 (*p* = 0.625) (Table 3). The mean change of the BPI‐DPN score in the two groups over 10 weeks is shown in Figure 2.

**TABLE 2:** Analysis of changes in BPI‐DPN, NRS‐11, and DSID (FAS population)

Variable	Pregabalin group				XF group			
	Baseline	10 weeks	*t*/*Z*	*p*	Baseline	10 weeks	*t*/*Z*	*p*
BPI‐DPN	52.03 ± 14.30	37.85 ± 17.23	−4.629	<0.001	42.44 ± 17.56	26.47 ± 22.22	−4.225	<0.001
NRS‐11	6.24 ± 1.23	4.41 ± 1.72	−4.182	<0.001	5.47 ± 1.13	3.56 ± 2.11	−4.187	<0.001
DSID	6.24 ± 1.72	4.18 ± 1.68	−4.067	<0.001	5.53 ± 1.93	3.47 ± 2.58	−4.070	<0.001

Abbreviations: BPI‐DPN, Brief Pain Inventory for Diabetic Peripheral Neuropathy; DSID, Daily Sleep Interference Diary; FAS, full analysis set; NRS‐11, numeric rating scale‐11; OHD, oral hypoglycemic drug; XF, Xiaoketongbi Formula; *t*, Student’s‐test statistic value; Z, Z‐test statistic value.

**TABLE 3:** Analysis of changes in primary and secondary outcomes (FAS population)

Variable	Pregabalin group	XF group	Difference (95% CI)	*t*/*Z*	*p*
BPI‐DPN					
Baseline	52.03 ± 14.30	42.44 ± 17.56	9.59 (1.83–17.34)	2.469	0.016
10 weeks	37.85 ± 17.23	26.47 ± 22.22	4.82 (1.76–21.01)	2.361	0.021
Absolute change	14.18 ± 14.08	15.97 ± 15.99	−1.79 (−9.09–5.50)	−0.491	0.625
NRS‐11					
Baseline	6.24 ± 1.23	5.47 ± 1.13	0.76 (0.19–1.34)	2.662	0.010
10 weeks	4.41 ± 1.72	3.56 ± 2.11	0.85 (−0.08–1.78)	1.827	0.072
Absolute change	1.82 ± 1.82	1.91 ± 1.93	−0.09 (−0.99–0.82)	−0.194	0.847
DSID					
Baseline	6.24 ± 1.72	5.53 ± 1.93	0.71 (−0.18–1.59)	1.592	0.116
10 weeks	4.18 ± 1.68	3.47 ± 2.58	0.71 (−0.35–1.76)	1.336	0.186
Absolute change	2.06 ± 2.16	2.06 ± 2.28	0.00 (−1.07–1.07)	0.000	1.000
PGIC 10 weeks	3.00 (3.00 ~ 4.00)	3.00 (2.00 ~ 4.00)		−1.070	0.285
Median nerve MNCV					
Left	−1.1 ± 3.6	−0.2 ± 2.2	−1.4 (−3.1, − 0.3)	−1.604	0.117
Right	−2.2 ± 4.1	0.7 ± 2.3	−2.9 (−4.7, − ‐1.0)	−3.044	0.004
Tibial nerve MNCV					
Right	0.1 ± 3.7	0.3 ± 2.7	−0.2 (−2.1, − 1.6)	−0.276	0.784
Left	−0.2 ± 4.2	0.7 ± 2.5	−1.0 (−2.9, − 1.0)	−1.003	0.321
Median nerve SNCV					
Left	−1.3 ± 7.6	−0.01 ± 4.4	−1.3 (−4.8, − 2.3)	−0.730	0.469
Right	−2.3 ± 6.4	0.8 ± 5.2	−3.1 (6.5, − 0.2)	−1.886	0.065
Sural nerve SNCV					
Right	−1.3 ± 9.1	1.6 ± 5.9	−2.9 (−7.7, − 2.0)	−1.201	0.237
Left	−2.1 ± 8.5	2.1 ± 5.1	−4.3 (−8.8, − 0.3)	−1.892	0.067

Abbreviations: BPI‐DPN, Brief Pain Inventory for Diabetic Peripheral Neuropathy; DSID, Daily Sleep Interference Diary; FAS, full analysis set; NRS‐11, numeric rating scale‐11; MNCV, motor nerve conduction velocity; PGIC, Patient Global Impression of Change; SNCV, sensory nerve conduction velocity; XF, Xiaoketongbi Formula.

### 3.3 The proportion of patients with a reduction of BPI‐DPN >50%

At week 10, 44.1% of patients (15/34) in the XF group and 20.6% of patients (7/34) in the pregabalin group reported a reduction of BPI‐DPN >50%. The between‐group difference was statistically significant (χ^2^ = 4.300, *p* = 0.038).

### 3.4 Changes in NRS‐11, DSID, and PGIC scores

After 10 weeks of treatment, NRS‐11 and DSID scores in both groups decreased compared with baseline levels (*P*_s_ < 0.001) (Table 2). However, there were no significant differences in absolute change of NRS‐11 and DSID scores between the two groups (Table 3). The difference in NRS‐11 score was −0.09 (95% CI: −0.99, 0.82; *p* = 0.847), and the difference in the DSID score was 0.00 (95% CI: −1.07, 1.07; *p* = 1.000). Mean changes of the NRS‐11 and DSID in the two groups over 10 weeks are shown in Figure 2. More significant differences were observed in PGIC scores; four patients felt “significantly improved” and eight patients felt “improved” in the XF group, whereas no patient felt “significantly improved” and only four felt “improved” in the pregabalin group after treatment. A greater number of patients felt “significantly improved” or “improved” in the XF group than in the pregabalin group (35.3% [n = 12/34] vs. 11.8% [4/34], χ^2^ = 4.005, *p* = 0.045).

### 3.5 Changes in NCV

The absolute change of the MNCV of the right median nerve was 0.7 ± 2.3 in the XF group and −2.2 ± 4.1 in the pregabalin group. The between‐group difference was −2.9 (95% CI: −4.7, −1.0), which was statistically significant (*p* = 0.004) (Table 3). The MNCV of the left median and bilateral tibial nerve and the SNCV of the median nerve and the sural nerve tended to increase in the XF group but decreased in the pregabalin group. There were no significant differences in absolute change between the groups (*P*_s_ > 0.05) (Table 3).

### 3.6 Safety

Neither group reported SAEs during treatment. There was no significant difference in the incidence of AEs between XF and pregabalin groups (11.8% [n = 4] vs. 5.9% [n = 2], *p* = 0.673).

Patients in the XF group complained of increased stool frequency without abdominal pain (n = 4). In contrast, dizziness (n = 1) and somnolence (n = 1) were reported in the pregabalin group. All enrolled patients followed the treatment plan and completed the study.

## 4 DISCUSSION

To our knowledge, this is the first randomized controlled trial to compare the efficacy and safety of CHMs with pregabalin in patients with PDN. Importantly, the blind and double‐dummy study design minimized potential bias to obtain robust results. Moreover, subjective measures, including indices to measure pain, sleep, and quality of life, and objective measures, such as NCV, were utilized to comprehensively and accurately evaluate treatment efficacy.

Pregabalin remains the mainstay of treatment for PDN. The analgesic effect of pregabalin observed in this study is consistent with results reported in the literature.^11^, ^12^ Many patients enrolled in this study experienced nerve pain, which seriously affected their lives and work. They previously underwent medical treatment, which was ineffective. In this study, after treatment for 10 weeks, 44.1% of patients (15/34) in the XF group and 20.6% of patients (7/34) in the pregabalin group reported a reduction of BPI‐DPN >50% (χ^2^ = 4.300, *p* = 0.038), which showed that XF could effectively provide pain relief in terms of PDN scores.

Patient quality of life, including sleep and mood, plays a vital role in treating PDN. It is widely thought that improving sleep quality and reducing anxiety and depression are essential to yield a therapeutic effect. Our findings suggest that XF positively affected the patient's overall impression of the quality of life compared with pregabalin. A greater number of patients felt “significantly improved” or “improved” in the XF group than in the pregabalin group (*p* = 0.045).

NCV is the most sensitive and specific method for detecting DPN and is an objective measurement to evaluate nerve function. There is clear evidence that pregabalin exerts no significant effect on NCV in patients with PDN.^34^, ^35^, ^36^ In the present study, the NCV tended to decline in the pregabalin group after 10 weeks of treatment, suggesting that pregabalin may relieve pain symptoms but yielded no definite effect on improving nerve function, which is consistent with what is reported in the literature. However, we found that NCV tended to increase in the XF group. Although significant differences were not observed in all NCVs after treatment, the MNCV of the right median nerve increased in the XF group but was reduced in the pregabalin group (*p* = 0.004). This finding suggests that XF could improve the symptoms of neuropathy and nerve function with an increase in the NCV. The results of our study provide evidence of the potential of CHMs to increase NCV, which warrants further study.

Safety plays an important role in drug evaluation. It is widely acknowledged that the main side effects of pregabalin include dizziness, lethargy, peripheral edema, and weight gain, and suicide attempts have been reported in severe cases.^37^, ^38^, ^39^ The adverse reactions of pregabalin are often more obvious in elderly patients, exhibiting a dose‐dependent effect. In the present study, it was found that pregabalin could relieve pain and improve sleep quality; however, adverse reactions were also observed, such as dizziness and somnolence. No AEs were observed in the XF group, except for increased stool frequency. In this respect, stools are often harder in PDN patients (hard, normal, and loose stools accounted for 42.6%, 48.5%, and 8.8% of patients in this study, respectively). Constipation is widely acknowledged as a significant concern for PDN patients. We found that patients taking XF have smoother stools without gastrointestinal symptoms, such as nausea, vomiting, abdominal pain, and diarrhea. After 10 weeks of treatment, 29.4% of patients (10/34) in the XF group vs. 17.6% of patients (6/34) in the pregabalin group experienced constipation relief; this may be attributed to the peach kernels and *R. officinale* contained in XF, which have a laxative effect and may account for the therapeutic effect of XF to a certain extent, warranting further study. In addition, according to Chinese regulations,^40^, ^41^ as a traditional Chinese medicine compound preparation, XF can be directly used in clinical practice without the need for preliminary safety clinical trials.

In interpreting the results of our study, some limitations should be taken into consideration. First, the sample size of the study was small, and no stratified randomization was used, which may have led to randomization imbalances contributing to the higher baseline values in the pregabalin and XF groups for BPI‐DPN (52.03 ± 14.30 vs. 42.44 ± 17.56) and NRS‐11 (6.24 ± 1.23 vs. 5.47 ± 1.13) scores. No significant differences in general patient characteristics, DSID, and PGIC were found prior to treatment. Accordingly, the absolute changes in BPI‐DPN, NRS‐11, and DSID were compared between the two groups to reduce the impact of baseline variations. Moreover, the study did not include a double‐blind control, and patients were enrolled from only one center. Given that PDN is a recurring disease, a short‐term effect cannot indicate a long‐term prognosis. In addition, the patients’ anxiety and depression status were not evaluated. Lastly, this is an exploratory study, and the frequency of XF administration in this study was once daily. At present, no study has comprehensively assessed the effects of different XF doses. Accordingly, further research should be conducted to test the robustness of our findings.

In conclusion, this is the first prospective randomized clinical trial in which the efficacy and safety of the CHM XF is compared to pregabalin in patients with PDN. We found that XF has an equivalent effect to pregabalin in reducing pain symptoms and is more effective in improving the quality of life in patients with PDN. In addition, XF has the potential to improve nerve function by increasing NCV and is well tolerated by PDN patients. Confirmatory trials will be required to investigate the full efficacy of XF.

## AUTHOR CONTRIBUTIONS

Q.Y.L., Z.J.L., and B.J.C. contributed to the study design, data curation, analysis and interpretation, and the manuscript drafting. Z.J.L. and G.J.F. obtained funding and conceived, organized, and supervised the study. Q.S.L., L.Y.W., L.L.L. collected the data. W.W.O.Y. and Z.H.W. performed the statistics analysis. A.X.L., Y.W.L., J.Y.L., and Y.Y.L. contributed to data acquisition. All authors were involved either in the drafting or the revision of the manuscript. All authors have approved the final version of the manuscript.

## FUNDING INFORMATION

This work was supported by the Specific Research Fund for TCM Science and Technology of Guangdong Provincial Hospital of Chinese Medicine (No. YN2016ML03), Guangzhou Civil Affairs Bureau Planned Project of Science and Technology (No. 2021MZK29), and Zhaoyang Talent Project of Guangdong Provincial Hospital of Chinese Medicine (No. ZY2022YL20). The funders/sponsors had no role in the design and conduct of the study; collection, management, analysis, and interpretation of the data; preparation, review, or approval of the manuscript; and decision to submit the manuscript for publication.

## CONFLICTS OF INTEREST

No potential conflicts of interest relevant to this article were reported.
